# [Retracted] Combination of magnetic resonance imaging and targeted contrast agent for the diagnosis of myocardial infarction

**DOI:** 10.3892/etm.2026.13171

**Published:** 2026-04-27

**Authors:** Jiangjun Qin, Shuchang Zhou, Zhiwei Li, Yinan Chen, Qun Qin, Tao Ai

Exp Ther Med 16:3303-3308, 2018; DOI: 10.3892/etm.2018.6600

Following the publication of this paper, it was drawn to the Editor’s attention by a concerned reader that various of the images showing the morphological analysis of myocardial infarction in Fig. 2 were strikingly similar to data that had already appeared in different form in Figs. 2 and 4 of another article written by different authors at different research institutes, which had been published two years previously in the journal *Circulation: Heart Failure*.

In view of the fact that the abovementioned data had already apparently been published prior to the receipt of this article at *Experimental and Therapeutic Medicine*, the Editor has decided that this paper should be retracted from the Journal. The authors were asked for an explanation to account for these concerns, but the Editorial Office did not receive a reply. The Editor apologizes to the readership for any inconvenience caused.

